# Some Aspects of Intestinal Obstruction

**Published:** 1908-07-11

**Authors:** 


### SOME ASPECTS OF INTESTINAL OBSTRUCTION.

### OBSTRUCTION DUE TO DISEASE OF THE INTESTINAL WALL OTHER THAN CARCINOMA.

Sarcoma of the intestine is a rare condition which affects the small more often than the large intestine. The growth, which is of the small round-celled variety, starts in the submucosa and attacks the muscular, but not usually the serous, coat of the gut. Macroscopically the sarcoma produces a diffuse infiltration of the gut wall. The clinical picture differs largely from that presented by carcinoma. Enterostenosis is rarely produced by the sarcoma *per se*. On the contrary, the affected gut is dilated in an aneurysmal fashion, since the growth destroys the muscles of the bowel wall. Obstruction, if it occurs, is generally due to such a cause as the sinking of a coil of dilated bowel so as to form a kink, or a condition of ileus may follow on paralysis of a section of the wall of the bowel.

In these cases, as opposed to carcinoma, the local condition is quite subordinate to the general impairment of health caused by the disease, the sarcomatous "cachexia," and the duration of life from the date of onset of symptoms rarely exceed nine months. In carcinoma, on the other hand, the average duration of life is twice as long as this.

Obstruction due to innocent tumours springing from the bowel wall is rare, but does occasionally occur. Clinically, the tumours may occur as flat tumours, connected to the bowel by a broad base or as polypoid excrescences with a pedicle. Their morphological classification is based upon the nature of the tissue from which they originate. Thus we recognise tumours derived from (i), epithelium—adenomata; (ii), connective tissue fibromata, lipomata and papillomata; (iii), the muscular coat-myomata, and (iv), the blood-vessels—angeiomata.

A very short description of their individual characteristics will suffice. The adenomata are the commonest; they originate from the crypts of Lieberkühn, and present on section a number of acini lined with columnar cells. They vary in size from a pea to a walnut, and they differ from carcinoma in being frequently multiple, but are very apt to degenerate into that condition. The connective tissue tumours are most often lipomatous; a genuine fibroma, myoma or angeioma of the bowel wall is exceedingly rare.

Benign tumours may give rise to no symptoms during life and may attain a considerable size without their presence being suspected. Sir Prescott Hewett reported a case where a polypoid tumour of the ileum as large as a pear had caused no symptoms until it produced an intussusception. They occasionally give rise to a genuine chronic obstruction by more or less completely blocking the bowel. When this happens the differential diagnosis of the cause is exceedingly difficult. The growth may sometimes be felt by the finger in the rectum, since benign tumours affect most commonly the lower end of the alimentary canal. But the only feature of any real diagnostic value is the fact that they tend to cause a hæmorrhage from the bowel which is more profuse than that associated with other conditions leading to stenosis.

Cicatricial stricture depends upon the contracting of a cicatrix, consequent upon loss of substance by ulceration of the inner coats of the bowel. There is hardly any form of intestinal ulcer which has not been credited somewhere or other in medical literature with the formation of a subsequent cicatricial stricture. If the accounts of these conditions are critically examined, it will be seen that the evidence connecting the stricture with the ulcer is rarely very convincing, and it may be said at once, in the case of certain ulcers, that even if the chain of evidence is complete the occurrence is so rare as to be of little, if any, clinical importance. In this category may be classed the strictures said to follow duodenal, syphilitic, typhoid, and follicular ulcers of the bowel. Dysenteric ulcer used to be regarded in old times as a common precursor of stenosis, until Woodward pointed out that in an enormous number of cases of dysentery which occurred in the American war of secession he was unable to convince himself that stricture due to cicatrisation followed in any single case.

Any form of ulceration predisposes, of course, to carcinoma, and in this way stricture may be brought about. But a case of obstruction due to genuine cicatrisation of any of the ulcers mentioned above is exceedingly rare.

There is, however, one form which must be excluded from this category, and that is the tuberculous ulcer. This can be easily understood. The tuberculous ulcer of the bowel is a common affection. It takes its origin in a Peyer's patch and tends to spread transversely round the bowel in the direction of the blood-vessels. This explains the comparative frequency of intestinal stenosis after tuberculous ulcer. In many of the cases reported, the strictures were found post-mortem to be multiple. The symptoms are those of typical chronic intestinal obstruction, but the diagnosis is difficult. It will rest mainly on the demonstration of another tuberculous focus in the patient—*e.g.*, pulmonary phthisis—and of tubercle bacilli in the dejecta.

While on the subject of intestinal tuberculosis, reference may be made to a tuberculous formation in the cæcum, to which attention has been directed in recent years by Billroth. This is a tumour-like growth which simulates, clinically and macroscopically, malignant disease of that organ. Histologically it consists of a small-celled infiltration of the cæcal wall, in which giant cells and, occasionally, tubercle bacilli, can be demonstrated. It causes an intestinal stenosis at the ileo-cæcal valve, whose symptoms cannot be clinically differentiated from those of any other form of enterostenosis. The tumour formed in the right iliac fossa is said to differ from carcinoma of the cæcum in having no edges and in shelving off indefinitely into the ascending colon, whereas a carcinoma terminates more or less abruptly. The presence of tubercle bacilli in the stools or the existence of another tuberculous focus will, as in the case of tuberculous ulcer, strengthen the diagnosis.

